# Studies of Non-Protective Autophagy Provide Evidence that Recovery from Therapy-Induced Senescence is Independent of Early Autophagy

**DOI:** 10.3390/ijms21041427

**Published:** 2020-02-20

**Authors:** Tareq Saleh, Liliya Tyutyunyk-Massey, Nipa H. Patel, Emmanuel K. Cudjoe, Moureq Alotaibi, David A. Gewirtz

**Affiliations:** 1 Department of Basic Medical Sciences, Faculty of Medicine, The Hashemite University, Zarqa 13133, Jordan; tareq@hu.edu.jo; 2 Massey Cancer Center, Goodwin Research Laboratories, Virginia Commonwealth University, Richmond, VA 23298, USA; tyutyunykmals@vcu.edu (L.T.-M.); patelnh3@vcu.edu (N.H.P.); 3 Department of Pharmacology and Toxicology and Medicine, Virginia Commonwealth University, Richmond, VA 23298, USA; 4 Department of Pharmacotherapy & Outcomes Science, Virginia Commonwealth University, Richmond, VA 23298, USA; cudjoeek@vcu.edu; 5 College of Pharmacy, King Saud University, Riyadh 11451, Saudi Arabia; mralotaibi@ksu.edu.sa

**Keywords:** senescence, autophagy, cancer, chemotherapy, radiotherapy, proliferative recovery

## Abstract

Autophagy and senescence, predominant responses that may dictate cell fate after chemotherapy or radiation, often occur in tandem. Cells in states of senescence and/or autophagy are frequently growth arrested. We have previously reported that tumor cells induced into senescence by therapy can re-emerge from the growth-arrested state, a phenomenon termed proliferative recovery. The current work shows that, while tumor cells collaterally induced into senescence and autophagy by etoposide, doxorubicin, or radiation undergo proliferative recovery, neither pharmacological nor genetic inhibition of early autophagy alter the extent of senescence or the ability of cells to recover from senescence. These findings confirm and extend our previous observations, essentially dissociating senescence from autophagy, and further indicate that re-emergence from senescence does not appear to be facilitated by or dependent on autophagy. Our results also provide additional evidence for the promotion of the non-protective form of autophagy by both chemotherapeutic drugs and radiation, which may complicate current efforts to inhibit autophagy for therapeutic benefit.

## 1. Introduction

Accelerated or premature senescence is a common tumor cell response to conventional cancer therapy [1,2]. Senescent tumor cells are growth-arrested and exhibit flat and hypertrophic cellular morphology, increased activity of the lysosomal senescence-associated β-galactosidase (SA-β-gal), epigenetic changes, as well as a genetic expression profile reflective of the senescence-associated secretory phenotype (SASP) [3]. Due to the durable nature of the senescent growth arrest, the use of senescence-inducing agents is considered an adventitious approach for cancer therapy based on the promotion of a static barrier against further tumor growth [4]. However, recent studies have strongly suggested that the senescent growth arrest precipitated by anticancer therapy [therapy-induced senescence (TIS)] is not terminal, and that a subpopulation of the senescent tumor cells can resume division, a process termed proliferative recovery [5,6]. Furthermore, the recovery from TIS was found to be permissive for the development of a more aggressive malignant phenotype [7,8,9]. Finally, the SASP has been shown to potentially promote tumor growth [10].

Based on these findings, we recently suggested that senescence could be one form of tumor dormancy, and, consequently, that recovery from senescence could contribute to disease recurrence [11]. It has also been suggested that macroautophagy (hereafter, autophagy) contributes to the maintenance of tumors in a dormant state [12]. Autophagy is a homeostatic process that involves lysosomal-dependent intracellular degradation of damaged organelles and misfolded proteins and scavenging of reactive oxygen species [13,14]. Autophagy and senescence tend to occur in parallel [15,16,17]; in a recent report, we demonstrated a linear relationship between autophagy and senescence induced by radiation in HCT-116 colorectal cancer cells [17]. A number of studies have investigated the putative, or potential, relationship between autophagy and senescence, generally concluding that, while autophagy may accelerate both oncogene-induced senescence and chemotherapy-induced senescence, senescence can and does occur even in the absence of autophagy [15,18,19]. However, one intrinsic limitation relating to studies of this relationship is that, when autophagy expresses its cytoprotective form, autophagy inhibition results in apoptotic cell death [20,21,22]; consequently, it becomes difficult to distinguish between the impact of autophagy inhibition on cell killing and the direct effects of autophagy inhibition on senescence.

To circumvent this limitation, the current studies were performed using three tumor cell lines and therapeutic modalities that induce what we have previously termed the non-protective form of autophagy [23]. By definition, when non-protective autophagy is inhibited, there is no increase in apoptotic cell death or alterations in drug or radiation sensitivity. Utilizing this approach, we were able to essentially dissociate autophagy from both senescence induction as well as recovery/escape from senescence. These studies therefore indicate that the generation of energy and metabolic precursors that are the hallmarks of autophagy do not appear to be required for the cells to enter into senescence arrest or facilitate subsequent proliferative recovery. Extrapolating these findings to the clinical impact of cancer therapy, we propose that autophagy (at least in its early stages) may not contribute to the capacity of tumor cells to enter a state of dormancy or to re-emerge from dormancy into an active reproductive state.

## 2. Results

### 2.1. Etoposide-Induced Autophagy does not Influence the Survival or Recovery of H460 NSCLC Senescent Cells

**Figure 1:**
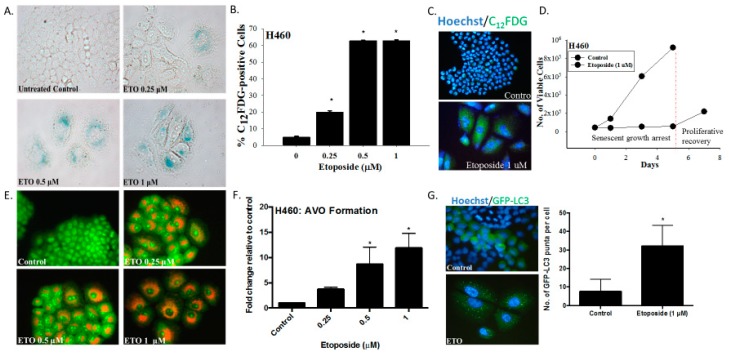
The induction of senescence and autophagy in H460 cells in response to etoposide. (**A**) Senescence-associated β-galactosidase staining of H460 cells exposed to etoposide (0.25, 0.5, or 1 µM) 48 h after drug removal (20x objective). (**B**) Quantification of senescence based on C_12_FDG staining of H460 cells followed by fluorescence-activated cell sorting (FACS) analysis. (**C**) Widefield fluorescent microscopy showing C_12_FDG staining. Nuclei stained with Hoechst 33342 (20x objective). Staining and analysis were performed 48 h after drug removal (**D**) Growth arrest and proliferative recovery of H460 cells exposed to etoposide (1 µM) for 24 h (day 0). (**E**) Fluorescence microscopy showing concentration-dependent increase in acridine orange-stained vacuoles induced by 0.25, 0.5, and 1 µM etoposide (20x objective). (**F**) Quantification of acidic vesicular organelles (AVOs) by FACS analysis in response to increasing concentrations of etoposide. (**G**) Fluorescence microscopy showing increased GFP-LC3 puncta in response to etoposide (1 µM) exposure. Imaging performed 48 h after drug removal. (20x objective). Quantification of GFP-LC3 puncta formation in response to etoposide exposure. Results presented were from three independent experiments, unless otherwise indicated. *p < 0.05 compared to untreated controls.

Our first series of studies examined the induction of senescence and autophagy in H460 non-small cell lung cancer cells exposed to etoposide. Within three days after initiation of drug treatment, H460 cells exhibited numerous features collectively indicative of senescence, specifically a flattened and enlarged appearance with abundant granulation and histochemical staining for SA-β-galactosidase (SA-β-gal) activity (Figure 1A). Using an established C_12_FDG (a fluorescent SA-β-gal surrogate) fluorescent labeling procedure of flow cytometric analysis coupled with fluorescent microscopy [24], quantification of H460 cells expressing SA-β-gal activity was determined over a range of etoposide concentrations (Figure 1B). Figure 1C shows C_12_FDG staining indicative of drug-induced senescence by fluorescence microscopy. Figure 1D indicates that H460 cells exposed to etoposide were growth arrested for at least five days, consistent with the induction of TIS. This senescent growth arrest was followed by proliferative recovery between 5 and 7 days post-drug exposure, in agreement with our previously reported findings of the capacity of a subpopulation of senescent tumor cells to regain proliferative capacity [6].

As would have been anticipated based on the fact that etoposide has previously been shown to promote autophagy in the A549 and U1810 NSCLC cells [25], autophagy was also evident in the H460 cells exposed to etoposide, as indicated by the increased formation of acridine orange-stained acidic vesicular organelles (Figure 1E, with quantification in Figure 1F). The induction of autophagy was confirmed based on the increased formation of GFP-LC3 puncta, indicative of autophagosome formation (Figure 1G).

**Figure 2:**
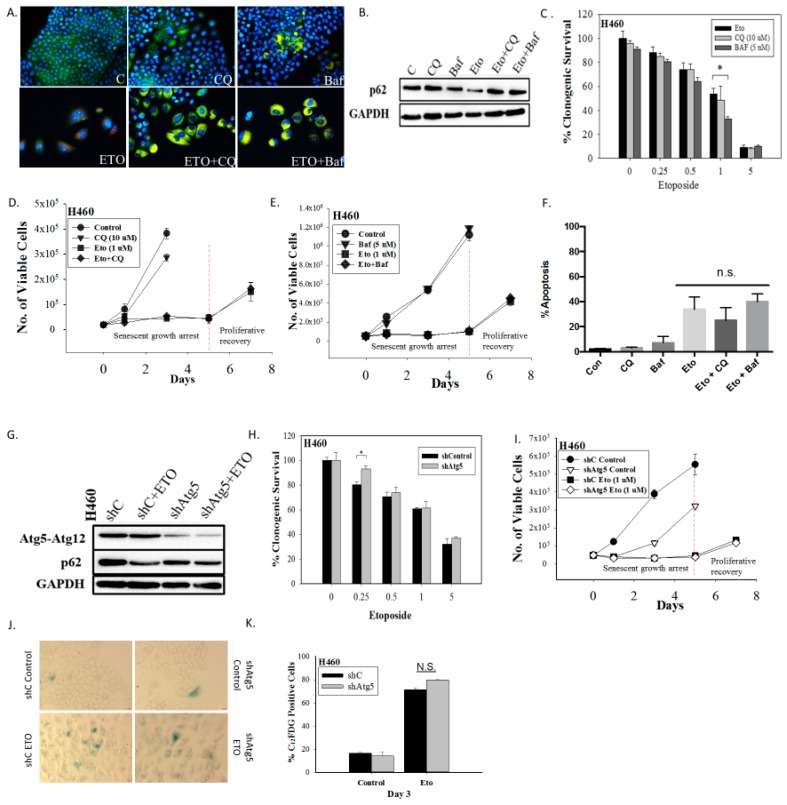
Inhibition of autophagy does not interfere with the induction or the recovery from senescence in H460 cells exposed to etoposide. (**A**) Fluorescence microscopy showing failure of lysosomal acidification following chloroquine (CQ, 10 µM) or bafilomycin A1 (Baf, 5 nM) co-treatment with etoposide (ETO, 1 µM). Cells were pretreated with CQ and Baf followed by an additional 24 h with etoposide. Images were taken 48 h after drug removal. Nuclei stained with Hoechst 33342 (20x objective). (**B**) Western blot showing autophagy blockade by CQ (10 µM) and Baf (5 nM) based on levels of p62/SQSTM1 (**C**) Clonogenic survival assay showing influence of CQ (10 µM) or Baf (5 nM) on sensitivity of H460 cells to etoposide. Cells were pretreated with CQ or Baf for 3 h followed by co-treatment with etoposide for 24 h. Colonies were counted 7 days following removal of drugs and replacement with fresh medium. Bars represent mean survival ± SD relative to untreated controls (α = 0.05/3, * *p* < 0.016). (**D**) and (**E**) Temporal response to etoposide in H460 cells after pharmacological autophagy inhibition. Viable H460 cell number was determined at the indicated days following etoposide exposure in combination with 10 µM CQ (D) or 5 nM Baf (**E**). (**F**) Assessment of apoptosis 48 h after drug removal (n.s. = no significant difference). (**G**) Western blot following short hairpin RNA (shRNA)-mediated knockdown of Atg5. (**H**) Clonogenic survival assay comparing sensitivity of shControl and shAtg5 H460 cells in response to multiple etoposide concentrations. Bars represent mean survival ± SD relative to untreated controls (α = 0.05/3, * *p* < 0.016). (**I**) Temporal response to etoposide in shControl H460 cells and H460 cells with knockdown of Atg5. (**J**) Etoposide-induced senescence in both autophagy-proficient and autophagy-deficient H460 cells by staining for SA-β-gal activity (20x objective). (**K**) Percent senescence based on C_12_FDG staining at day 3 post-etoposide exposure in shControl cells and shAtg5 cells. Results presented were from three independent experiments unless otherwise indicated.

Autophagy has historically been considered a survival response under conditions of nutrient deprivation or hypoxia as well as a process that facilitates tumor growth and serves as a mechanism of resistance to therapy [26,27,28,29]. Consequently, we hypothesized that autophagy could serve to maintain metabolic homeostasis in the senescent tumor cells and might thereby be necessary for maintenance of the senescent state. To determine the potential involvement of etoposide-induced autophagy in maintaining senescence in the H460 cells, autophagy was suppressed using both pharmacological and genetic strategies applied early and followed by exposure to etoposide. The impact on cell viability was then monitored. H460 cells were pretreated for 3 hours with the autophagy inhibitors chloroquine (CQ, 10 µM) or bafilomycin A1 (Baf, 5 nM) followed by 24 hours of exposure to etoposide in the presence of the CQ or Baf. Exposure of H460 cells to the lysosomotropic agents CQ and Baf resulted in failure of lysosomal acidification [30,31], which is reflected by the yellow staining of vacuoles by acridine orange (Figure 2A); autophagy inhibition was confirmed by decreased degradation of p62/SQSTM1 in the presence of CQ or Baf in etoposide-treated cells (Figure 2B). The minimal effect of CQ and Baf on p62/SQSTM1 levels in etoposide-untreated cells is likely reflective of low basal levels of autophagy.

Inhibition of autophagy did not alter sensitivity to etoposide, as determined by clonogenic survival assays (Figure 2C) (except moderately with Baf at 1 µM etoposide), suggesting that the etoposide-induced autophagy was exhibiting a non-protective function and that the autophagy did not significantly contribute to the survival of senescent H460 cells [23]. This conclusion was further supported by the fact that early pharmacological autophagy inhibition did not alter growth arrest induced by etoposide, etoposide-induced apoptosis, or the proliferative recovery from etoposide-induced growth arrest and senescence (Figure 2D–F). Similar outcomes were evident when autophagy was inhibited by silencing Atg5-Atg12 (Figure 2G–K). Figure 2G presents a Western blot showing the knockdown of Atg5 and the consequent interference with etoposide-induced degradation of p62/SQSTM1. Figure 2H shows that silencing of *Atg5* did not significantly decrease the viability of senescent H460 cells (a small decrease in sensitivity was evident at the 0.25 µM concentration). As was the case with CQ and Baf, genetic autophagy inhibition failed to alter the extent of senescence (Figure 2J,K), the profile of growth arrest (Figure 2I), or the capacity of senescent tumor cells to undergo proliferative recovery (Figure 2I).

### 2.2. Doxorubicin-Induced Autophagy does not Influence the Survival or Recovery of 4T1 Breast Tumor Senescent Cells

The experimental data presented above provide another example of the non-protective form of autophagy induced by chemotherapy and further indicate that, while autophagy and senescence are induced collaterally by chemotherapy in tumor cells, autophagy is not necessary for the maintenance of senescence and does not appear to play a role in the recovery from senescence. In order to demonstrate that these findings are not limited to one therapeutic agent or experimental tumor cell line, similar studies were performed in 4T1 murine breast tumor cell lines exposed to doxorubicin (Dox). Here, it should be noted that we and our collaborators have previously identified the non-protective form of autophagy in response to radiation in the 4T1 cells [32].

**Figure 3:**
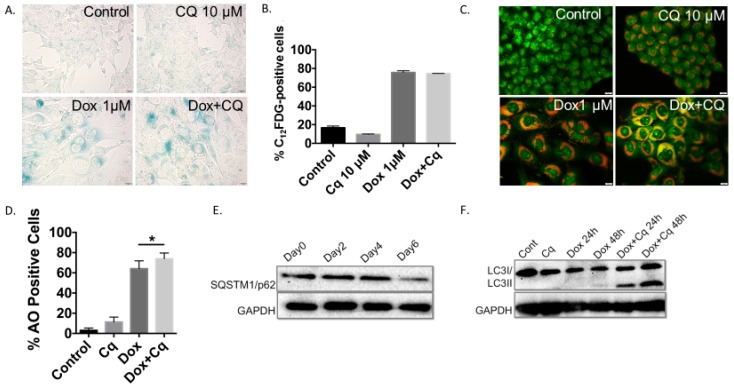
The induction of senescence and autophagy in 4T1 cells in response to doxorubicin (**A**) Induction of senescence determined by elevated senescence-associated β-galactosidase staining in 4T1 cells treated with 1 µM doxorubicin (Dox) for 2 hours (20x objective). (**B**) Quantification of senescence by FACS analysis based on C_12_FDG staining of 4T1 cells. (**C**) Fluorescence microscopy images showing an increase in acridine orange staining in cells treated with Dox (1 µM). 4T1 cells were pre-treated with CQ (10 µM) for 3 hours followed by 2 hour exposure to Dox with or without CQ (20x objective). (**D**) Quantification of autophagic cells by FACS analysis in response to Dox treatment showing accumulation of acridine orange (AO) positive cells that is further enhanced by inhibition of acidic degradation by CQ. (**E**) Induction of autophagy confirmed by Western Blot showing degradation of p62/SQSTM1 protein following a 2 h exposure to Dox (1 µM). (**F**) Blockade of autophagic flux by chloroquine resulting in accumulation of LC3BII protein. *p < 0.05 compared to untreated controls.

**Figure 4:**
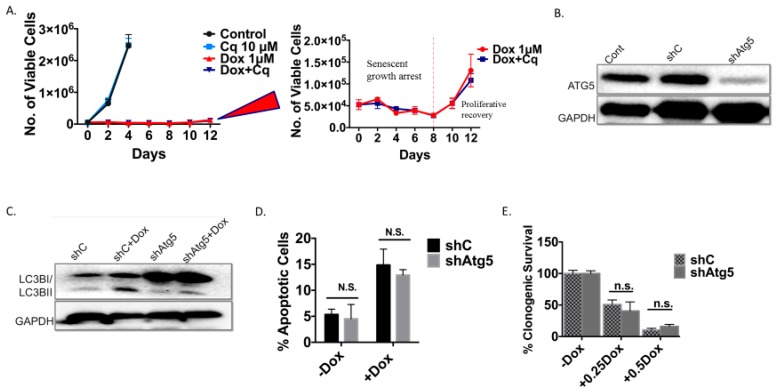

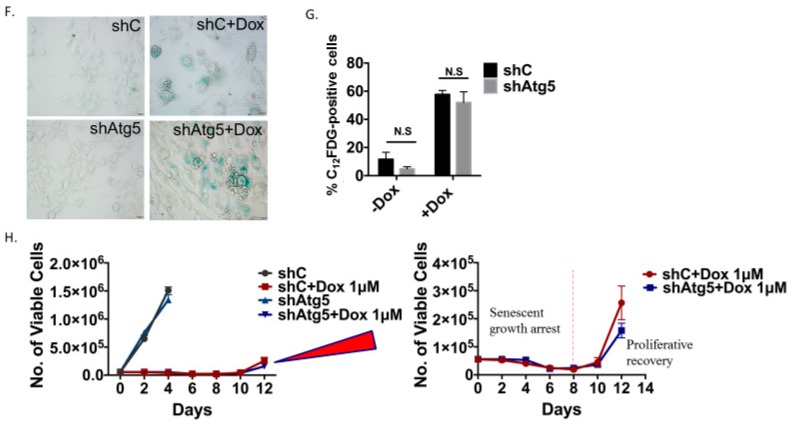
Inhibition of autophagy does not interfere with the induction or the recovery from senescence in 4T1 cells exposed to doxorubicin (**A**) Temporal response to Dox (1 µM) in 4T1 cells after pharmacological inhibition of autophagy with CQ. Viable cell number was determined at the indicated time points after treatment with Dox in combination with CQ (10 µM). Adjoining figure shows expanded scale for lower portion of the graph. (**B**) Knockdown of Atg5 in 4T1 cells confirmed by Western blot. (**C**) Western blot of Atg5 deficient 4T1 exposed to Dox showing conversion of LC3BI to LC3BII in WT cells, accumulation of LC3BI with decreased LC3BII in autophagy deficient cells. (**D**) Assessment of apoptosis by FACS analysis 24 hours post-treatment with Dox using APC/7AAD [Annexin 5/propidium iodide (PI) equivalent] dye. Genetic inhibition of autophagy did not result in increased apoptosis. (**E**) Colony formation assay comparing clonogenic survival of shControl and shAtg5 4T1 cells in response to multiple Dox concentrations. (**F**) Induction of senescence determined by SA-β-gal staining in shControl and shAtg5 4T1 cells treated with 1 µM Dox (20x objective). (**G**) Percent senescence based on quantification of C_12_FDG staining 48 hours after exposure of shControl cells and shAtg5 cells to Dox. (**H**) Temporal assessment of growth arrest followed by proliferative recovery in shControl and shAtg5 4T1 cells. Viable cell number was determined at the indicated time points after treatment with doxorubicin. Adjoining figure shows expanded scale for lower portion of the graph. (n.s. = no significant difference).

Figure 3 shows the collateral induction of senescence and autophagy in 4T1 cells by exposure to Dox. As above, senescence induction was analyzed based on β-galactosidase staining (Figure 3A) and quantification by flow cytometry (Figure 3B), while autophagy was detected based on acridine orange vacuole formation and quantification (Figure 3C,D) and confirmed by Western blotting showing p62/SQSTM1 degradation (Figure 3E). Autophagy inhibition was shown by increased accumulation of phagosomes resulting when lysosomal acidification was blocked by chloroquine (Figure 3C,D) and further confirmed by the accumulation of LC3B II protein (Figure 3F). It is important here to emphasize that Dox exposure in these experiments was limited to 2 hours and that, in response, autophagy was induced rapidly (Figure 3F); however, completion of autophagy was delayed for up to 6 days following drug removal, as indicated by p62/SQSTM1 degradation (Figure 3E). Early pharmacologic inhibition of autophagy did not interfere with the induction of senescence in the 4T1 cells, as SA-β-gal staining was prominent under both conditions—cells exposed to Dox alone and cells exposed to the combination of Dox and CQ (Figure 3A). Furthermore, the percentage of C_12_FDG-positive population was essentially identical when quantified by flow cytometry for cells exposed to Dox alone or to the Dox and CQ drug combination (Figure 3B). Similar to the outcomes for H460 cells exposed to etoposide, 4T1 cells exposed to Dox entered into a prolonged growth arrest followed by proliferative recovery, a profile that was not altered with inhibition of autophagy by CQ (Figure 4A and adjoining expanded figure).

As was the case with the H460 cells induced into autophagy/senescence by etoposide, the autophagy induced by Dox in the 4T1 cells was also shown to be non-protective. Silencing of Atg5 (Figure 4B,C) did not lead to increased apoptosis (Figure 4D), did not result in decreased cell survival based on colony formation (Figure 4E), and did not compromise the induction of senescence (Figure 4F,G). Cells were also induced into a prolonged growth arrest followed by proliferative recovery after 10 days independent of the Atg5 status of the cells (Figure 4H and expanded adjoining figure).

### 2.3. Radiation-Induced Autophagy does not Influence the Survival or the Recovery of HCT116 Senescent Cells

While etoposide and doxorubicin are both chemotherapeutic agents and largely have the same cellular target, i.e., topoisomerase II, it was important to determine whether these observations had broader implications by interrogating an entirely different therapeutic moiety, ionizing radiation, albeit one that also acts largely through the promotion of DNA damage, as is also the case for doxorubicin and etoposide. These experiments were performed in the HCT-116 colorectal carcinoma cell line.

**Figure 5:**
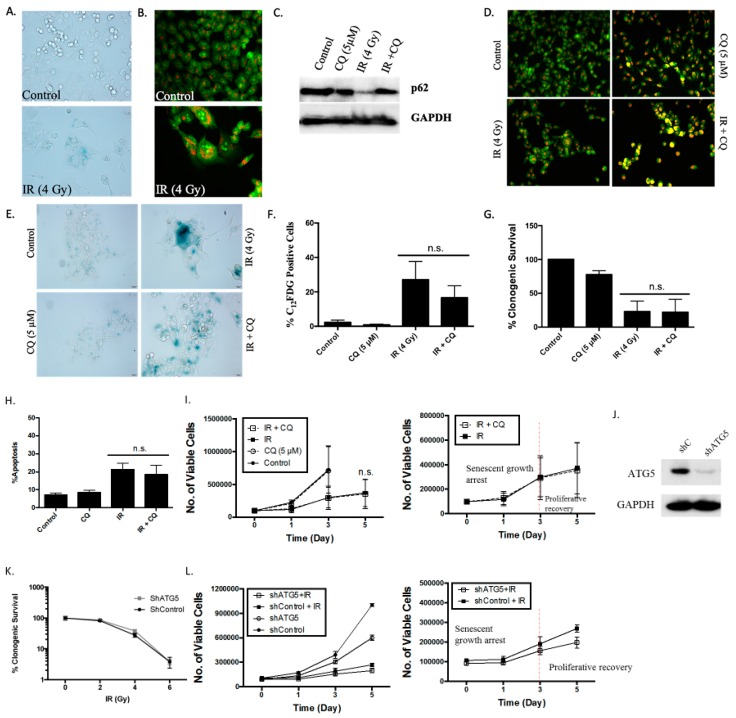
The induction of senescence and autophagy in HCT116 cells in response to radiation. (**A**) SA- β-galactosidase staining of HCT116 cells treated with 4 Gy radiation demonstrating induction of senescence (20x objective). (**B**) Fluorescent microscopy images of acridine orange staining 48 hours post-radiation (4 Gy). Increased acidic vesicle formation is visualized (20x objective). C-G. Cells were pre-treated with CQ (5 µM) 3 h prior to radiation (4 Gy) exposure. Media was replenished 24 h post-treatment. (**C**) Western blot analysis demonstrating autophagy blockade via p62 accumulation in cells pre-treated with CQ. (**D**) Acridine orange staining indicating blockade of lysosomal fusion in cells pre-treated with CQ (20x objective). (**E**) SA-β-galactosidase staining demonstrating increased SA-β-galactosidase activity in both cells exposed to radiation alone or pre-treated with CQ prior to radiation (20x objective). (**F**) SA-β-galactosidase activity was monitored by measuring C_12_FDG staining using flow cytometry. (**G**) Clonogenic survival assay showing radiation-induced growth inhibition in cells exposed to radiation (4 Gy) alone and in combination with CQ (5 µM). (**H**) Annexin 5/PI staining was used to assess apoptosis 48 h post-radiation [radiation (4 Gy) alone or with CQ (5 µM) pre-treatment]. Autophagy blockade did not alter radiation-induced apoptosis (*n* = 2). (**I**) Cells were treated with 4 Gy radiation alone or with CQ pre-treatment, and viable cell number was assessed via trypan blue exclusion on the indicated days. The adjoining figure shows the expanded scale for the lower portion of the graph. (**J**) Western blot demonstrating ATG5 knockdown. Autophagy blockade by shATG5 in the HCT-116 cells was established in our previous report [17]. (**K**) Clonogenic survival demonstrating dose-dependent reduction in both shControl and shATG5 knockdown cells. (**L**) Viable cell number was assessed in shControl and shATG5 HCT116 cells exposed to 4 Gy radiation. Representative curves of three independent studies are shown (*n* = 3). Results presented were from three independent experiments, unless otherwise indicated; n.s. represents no significant difference compared to radiation alone.

Figure 5A,B show the collateral induction of autophagy and senescence by ionizing radiation in the HCT-116 tumor cell lines. Autophagy and senescence induction were dose-dependent and occurred in parallel, as reported previously [17]. As in the studies presented in the H460 and/or the 4T1 cells, autophagy was pharmacologically inhibited early using CQ (Figure 5C). Specifically, HCT116 cells were pretreated with CQ (5 µM) for 3 hours before being irradiated and then maintained in culture medium for an additional 24 hours. Failure of lysosomal acidification in cells treated with CQ was demonstrated by the yellow staining of autophagic vacuoles (Figure 5D).

As shown in Figure 5E,F, senescence induced by radiation in the HCT-116 cells was not affected by autophagy inhibition. More specifically, inhibition of autophagy by CQ did not alter the sensitivity of HCT116 cells to radiation and did not promote radiation-induced apoptosis (Figure 5G,H), consistent with the radiation-induced non-protective autophagy observed in this experimental model. Figure 5I shows that HCT116 cells underwent growth arrest followed by proliferative recovery, evident 3 days post-radiation. Figure 5I further demonstrates that growth arrest and proliferative recovery profiles were virtually identical in HCT116 cells with and without pharmacological autophagy inhibition.

Finally, as was the case with chemotherapy in the H460 and 4T1 cell lines, genetic autophagy inhibition (silencing of ATG5, Figure 5J) did not influence radiation sensitivity (Figure 5K), growth arrest (Figure 5L), or proliferative recovery (Figure 5L).

## 3. Discussion

Both senescence and autophagy are established responses to stress resulting from DNA damage and oxidative injury. When apoptosis is not the predominant response to therapy, senescence represents a major determinant of cell fate, where cells remain in a growth-abrogated state as they maintain their metabolic activity [33]. In fact, it is feasible that senescence could represent one basis for tumor cells remaining dormant for prolonged periods of time [11]. Autophagy is also considered a “first or early responder” to cellular stress (in this case, DNA damage) resulting from the exposure to cancer chemotherapeutics or radiation [34,35,36]. It has been suggested that the regulatory pathways of both processes are intertwined [37,38,39], and it is clear that senescent cells develop abundant acidic vacuoles [40]. However, the relationship of the autophagic response to the induction and the maintenance of senescence does not appear to be consistent across the types of stimuli that promote these responses or the cell lines in which they have been studied [19].

**Figure 6:**
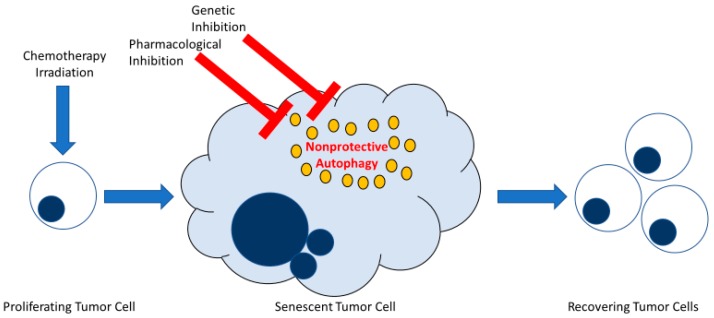
Early inhibition of non-protective autophagy does not interfere with recovery from therapy-induced senescence in tumor cells. Both senescence and autophagy are stress responses frequently induced in parallel in tumor cells exposed to DNA-damaging therapy. Therapy-induced senescence engenders a phase of stable growth arrest whereby a subpopulation of tumor cells can ultimately recover proliferative capacity. Autophagy induced in tumor cells in response to conventional chemotherapy or radiation takes on different functional outcomes. Of those, autophagy can be non-protective, meaning that when autophagy is blocked by pharmacological or genetic approaches, survival of tumor cells is not essentially affected. This figure illustrates how the inhibition of non-protective autophagy in therapy-induced senescent tumor cells does not interfere with tumor cell survival or the ability of these cells to recover from senescence.

While autophagy might have been anticipated to contribute to the maintenance of the metabolic integrity of the senescent tumor cells, early inhibition of autophagy induced following stress exposure did not appear to affect senescent cell survival. Furthermore, inhibition of autophagy, prior to and during drug or radiation exposure failed to prevent the tumor cell population from recovering and resuming growth. Consequently, while autophagy may potentially represent an intrinsic component of the senescent response elicited by cancer therapy, this current study indicates that autophagy plays a minor, if any, role in facilitating proliferative recovery in this system or interfering with the fate of senescent cells. The ability to reach this conclusion was facilitated by the fact that the autophagy was non-protective in function in all three experimental models, as illustrated in Figure 6 [23,41].

Although the bulk of the literature has focused on the cytoprotective function of autophagy [42,43], we and others have shown in a number of studies that interference with autophagy can fail to alter drug or radiation sensitivity or to promote apoptosis [44]. In this context, p53 appears to play an important regulatory role in determining the functional outcome of autophagy [45]. As would be expected, functional p53 is required for autophagy to exhibit a cytoprotective function, and, consequently, loss of p53 will either reduce the extent of the autophagic response or suppress its protective function. Accordingly, the non-protective function of radiation-induced autophagy was previously shown to be dependent on the cells being mutant or null in p53 [46]. However, in the current work, it is clear that autophagy induced by chemotherapy or radiation in the p53 wild-type H460 and HCT116 and p53 null 4T1 cells is also non-protective. These findings are consistent with a recent report by Eng et al. demonstrating non-protective autophagy induced by more than 30 chemotherapeutic drugs or radiation in the A549 NSCLC cell line [47]. In fact, previous studies addressing the involvement of autophagy in promoting an effective antitumor immune response following chemotherapy *in vivo* also demonstrated the non-protective form of autophagy (although this terminology had not been established at that time) [48]. Lastly, the induction of non-protective autophagy in senescent cells could be attributed to the accompanying lysosomal dysfunction resulting in failure of appropriate degradation of intra-lysosomal contents with possible alterations of the functional outcome of autophagy [49]. In agreement with our results, non-protective autophagy does not seem to be essential for the generation of senescent cells exhibiting dysregulated lysosomal biogenesis, as when when tumor cells are induced into senescence by CDK4/6 inhibitors [49].

The current study has a number of implications. One is that, since autophagy can be non-protective in tumor cells responding to chemotherapy or radiation, current clinical trials combining autophagy inhibition with cancer therapeutics are likely to be successful in demonstrating enhanced patient response only in those cases where the autophagy is actually protective [50]. A related conclusion is that the function of autophagy (whether it be protective or non-protective) cannot be uniformly linked to the p53 status of the tumor cell. Since senescence also appears to be a common response to therapeutics in solid tumors, interference with autophagy cannot be anticipated to prevent the prolonged growth arrest associated with senescence that may, in fact, serve to protect the tumor cells from therapy by preventing apoptosis. Finally, even in the case where the therapy-induced autophagy is protective and autophagy inhibition promotes cell killing, autophagy inhibition is unlikely to directly interfere with the generation of a residual, typically resistant, senescent tumor cell population. Consequently, if recovery from senescence proves to be one form of disease recurrence from dormancy, then autophagy inhibition may not directly interfere with disease recurrence. From an energetic perspective, it appears that senescent cells may not require, for their survival, the recycling of cellular organelles that is a central event in autophagy. It is therefore proposed that the elimination of tumor cells that survive the onslaught of chemotherapy or radiation by entering a state of senescence from which some tumor cells can ultimately escape will require the direct action of agents (e.g., senolytics) that are specific for this tumor cell population.

We recognize that a major limitation of the current work is the absence of studies to determine the contribution of autophagy to the survival of senescent tumor cells in tumor-bearing animals. Future studies will be designed to compare the tumor-initiating potential of autophagy-proficient and autophagy-deficient senescent tumor cells in an immunocompetent mouse model. In addition, experimental models will be developed where autophagy inhibition is sustained both in cell culture and in tumor-bearing animals in order to determine whether senescence and proliferative recovery might be compromised under conditions of prolonged autophagy suppression. Nevertheless, we believe that the current work serves to address a fundamental question relating to senescence and proliferative recovery (and possibly tumor dormancy and disease recurrence), which is whether the autophagy that generally accompanies senescence is necessary for senescence maintenance. In this regard, a recent publication identified a process of cellular cannibalism as potentially providing the energy necessary for senescent cell survival [51].

## 4. Materials and Methods

### 4.1. Cell Culture and Drug Treatment

The wild-type (WT) TP53 H460 cell lung cancer and HCT116 cell lines were generously provided by Dr. Richard Moran and Dr. Sarah Spiegel, respectively, at Virginia Commonwealth University. H460 and 4T1 cells were cultured in Dulbecco’s Modified Eagle Medium (DMEM), and HCT-116 cells were cultured in RPMI both supplemented with 10% (*v*/*v*) fetal bovine serum (Thermo Scientific, SH30066.03), 100 U/mL penicillin G sodium (Invitrogen, 15140–122), and 100 μg/mL streptomycin sulfate (Invitrogen, 15140–122). Cells were maintained at 37 °C under a humidified, 5% CO2 atmosphere at sub-confluent densities.

The ATG5-knocked down H460, HCT-116, and 4T1 variants were generated as follows: mission shRNA bacterial stocks for ATG5 were purchased from Sigma-Aldrich (TRCN00151963), and lentivirus generation was conducted in the HEK 293TN cells. Co-transfection was performed using lipofectamine (Invitrogen, 11668–019) with a packaging mixture of psPAX2 and pMD2.G constructs (Addgene, 12260, 12259). After 48 h, viruses shed into the media were collected and used to infect cells under ultrasonic centrifugation for 2 hours. Selection was performed in puromycin (Sigma-Aldrich, P8833) (1–2 μg/mL).

H460 LC3-GFP were generated previously [16]. In brief, cells were transfected with GFP-LC3 (Addgene, 22405) using lipofectamine (Invitrogen, 11668–019). Cells were fixed and fluorescence visualized using an Olympus inverted microscope (20X objective, Q-Color3™ Olympus Camera; Olympus, Tokyo, Japan). The number of LC3-GFP puncta for each cell was quantified.

At all etoposide (Sigma-Aldrich, E1383) concentrations, H460 cells were exposed to the drug-containing medium for 24 hours, followed by replacement with fresh medium. The 4T1 cells were exposed to doxorubicin (Tocris, 2252) for 2 hours, washed with phosphate-buffered saline (PBS), and supplemented with fresh medium. Incubation with chloroquine (CQ, 5 or 10 µM) or bafilomycin A1 (Baf, 5 nM) was utilized to interfere with lysosomal acidification and autophagosome/lysosome fusion. Cells were treated with the autophagy inhibitors for 3 hours prior to the subsequent exposure to etoposide, doxorubicin, or radiation and the autophagy inhibitor for an additional 24 hours to ensure blockade of autophagy. All drugs were protected from light during handling.

### 4.2. Growth Inhibition and Clonogenic Survival

Growth curves were generated based on cell viability as assessed by Trypan blue exclusion. Cells were seeded, treated (on day 0), and counted at the indicated time points following the removal of the drug from the medium. For clonogenic assays in H460 cells, cells were seeded, pre-treated with CQ (5 or 10 µM) or Baf (5 nM) for 3 h, then treated with etoposide (0.25, 0.5, 1.0, or 5 µM), doxorubicin (0.25, 0.5 µM), and radiation (2, 4, 6 Gy) alone or in combination with CQ or Baf. Drugs were then removed and replaced with fresh media after 24 hours. Cells were incubated for 7 days, then fixed with methanol, stained with crystal violet, and counted (ColCount, Discovery Technology International).

### 4.3. Analysis of Senescence and Autophagy by Flow Cytometry and Microscopy

All of the flow cytometry analyses were performed using BD FACSCanto II and BD FACSDiva software at the Virginia Commonwealth University Flow Cytometry Core Facility. For C_12_FDG (Life Technologies, D2893) and acridine orange analyses, 10,000 cells per replicate within the gated region were analyzed. Three replicates for each condition were analyzed in each independent experiment. Labeling procedures, gating, and analysis followed our previously published protocols with minor adjustment for the tested cell line [15,16,17]. To measure acidic vesicle formation, cells were stained with 1 μg/mL acridine orange for 20 min at 37 °C, washed with PBS, and visualized under a fluorescent microscope (20X objective, Q-Color3™ Olympus Camera; Olympus, Tokyo, Japan) or quantified using flow cytometry. For β-galactosidase and C_12_FDG staining, β-galactosidase labeling was performed as previously described by Dimri et al. [52] and in our previous publications [15,16,17]. Phase contrast images were taken using an Olympus inverted microscope (20X objective, Q-Color3™ Olympus Camera; Olympus, Tokyo, Japan). The C_12_FDG staining protocol was adopted from Debacq-Chainiaux et al. [24].

### 4.4. Western Blotting

Western blotting was performed as previously described [16]. Primary antibodies were used at a 1:1000 dilution except for GAPDH (1:2000-1:8000 dilutions). Primary antibodies: SQSTM1/p62 (BD Biosciences, 610497), ATG5 (Cell Signaling Technology, 2630), LC3B (Cell Signaling Technology, 3868).

### 4.5. Analysis of Apoptotic Cell Death

Apoptosis was monitored utilizing annexin-V-FITC/propidium iodide (PI) staining. H460 and HCT116 cells were stained 48 h post-treatment according to manufacturer protocol (Annexin V-FITC Apoptosis Detection Kit; BD Biosciences, 556547), and fluorescence was measured utilizing flow cytometry. Apoptosis in 4T1 cells was measured utilizing APC/annexin V-FITC with 7-AAD staining 24 h post-drug removal according to manufacturer protocol (APC/annexin V-FITC with 7-AAD Apoptosis Detection Kit: BioLegend, 640930). Fluorescence was measured using flow cytometry. All of the flow cytometry analyses were performed using BD FACSCanto II and BD FACSDiva software at the Virginia Commonwealth University Flow Cytometry Core Facility. For annexin-V-FITC/PI analysis and APC/annexin-V-FITC with 7-AAD analysis, 10,000 cells per replicate within the gated region were analyzed. Three replicates for each condition were analyzed in each independent experiment.

### 4.6. Statistical Analysis

GraphPad Prism 5.0 software was utilized to conduct statistical analysis. Data are shown as mean ± SEM from at least three separate experiments unless indicated otherwise. Statistical comparisons between groups were assessed via one-way ANOVA followed by Bonferroni post-hoc test and two-tailed *t* tests; *p*-value < 0.05 was considered statistically significant.

## 5. Conclusions

Taken together, the current studies indicate that neither the induction of senescence, the maintenance of senescence, nor the recovery from senescence appear to be dependent on the energetics and the metabolic precursor generation associated with the promotion of autophagy that occurs in response to anticancer therapy. These observations are more likely to be true when the therapy-induced autophagy exhibits a non-protective function, where inhibition of therapy-induced autophagy does not significantly affect the survival of tumor cells. These observations represent a foundation for further studies to elucidate the precise cellular mechanism(s) that are associated with maintaining the survival of senescent tumor cells and/or facilitating their potential recovery from a state of dormancy.
